# Risk of potential hepatotoxicity from pirfenidone or nintedanib in patients with idiopathic pulmonary fibrosis: results of a retrospective analysis of a large insurance database in Taiwan

**DOI:** 10.3389/fphar.2024.1309712

**Published:** 2024-02-07

**Authors:** Kuang-Ming Liao, Chung-Yu Chen

**Affiliations:** ^1^ Department of Internal Medicine, Chi Mei Medical Center, Chiali, Taiwan; ^2^ Master Program in Clinical Pharmacy, School of Pharmacy, Kaohsiung Medical University, Kaohsiung, Taiwan; ^3^ Department of Pharmacy, Kaohsiung Medical University Hospital, Kaohsiung, Taiwan; ^4^ Department of Medical Research, Kaohsiung Medical University Hospital, Kaohsiung, Taiwan

**Keywords:** idiopathic pulmonary fibrosis, nintedanib, pirfenidone, drug-induced liver injury, hepatotoxicity

## Abstract

**Background:** A growing population of individuals diagnosed with idiopathic pulmonary fibrosis (IPF) are receiving treatment with nintedanib and pirfenidone. The aim of our study was to assess the incidence of drug-induced liver injury (DILI) associated with the use of pirfenidone and nintedanib in patients with IPF in Taiwan.

**Methods:** We collected a cohort of adult patients diagnosed with IPF between 2017 and 2020. The research outcomes involved assessing the incidence of DILI in patients treated with nintedanib or pirfenidone. Poisson regression analysis was employed to estimate incidence rates, with and without adjustments for covariates, to calculate and present both unadjusted and adjusted incidence rate ratios (IRRs).

**Results:** The risk of DILI was greater in patients who received nintedanib than in those who received pirfenidone during the 1-year follow-up. Patients treated with nintedanib exhibited a heightened risk of DILI based on inpatient diagnoses using specific codes after adjusting for variables such as gender, age group, comorbidities and concomitant medications, with an adjusted incidence rate ratio (aIRR) of 3.62 (95% confidence interval (CI) 1.11–11.78). Similarly, the risk of DILI was elevated in patients treated with nintedanib according to a per-protocol Poisson regression analysis of outcomes identified from inpatient diagnoses using specific codes. This was observed after adjusting for variables including gender, age group, comorbidities, and concomitant medications, with an aIRR of 3.60 (95% CI 1.11–11.72).

**Conclusion:** Data from postmarketing surveillance in Taiwan indicate that patients who received nintedanib have a greater risk of DILI than do those who received pirfenidone.

## Introduction

Idiopathic pulmonary fibrosis (IPF) is an incurable and progressive lung condition characterized by irreversible lung fibrosis and unfavorable prognosis. (Bjoraker et al., 1998). This fatal disease has a previously reported median survival of 3–6 years before effective drug therapy (Schwartz et al., 1994). Previous studies have shown that IPF results from repeated epithelial cell damage and activation. Furthermore, alveolar epithelial cells activate, proliferate and migrate and trigger mesenchymal cells to mirror abnormal wound repair, form fibroblastic and myofibroblastic lesions and excessively accumulate in the extracellular matrix (Selman and Pardo, 2006).

Nintedanib and pirfenidone have been shown to be effective and safe and have been licensed for treating IPF. Pirfenidone (5-methyl-1-phenyl-2-[1H]-pyridone) combines anti-inflammatory, antifibrotic and antioxidant therapeutic effects to reduce pulmonary fibrosis, further decreasing the rate of lung function impairment and increasing survival time (Taniguchi et al., 2010). Nintedanib is a small molecule tyrosine kinase inhibitor that was developed with the aim of blocking the vascular endothelial growth factor pathway (Hilberg et al., 2008). Nintedanib has antifibrotic and anti-inflammatory effects *via* interference with fibrosis processes through the use of platelet-derived growth factor receptor (PDGFR) α and ß, vascular endothelial growth factor receptor (VEGFR) and fibroblast growth factor receptor (FGFR) (Wollin et al., 2015).

A randomized placebo-controlled trial showed that nintedanib is effective in patients with IPF, improves quality of life, reduces acute exacerbations, and significantly reduces the rate of forced vital capacity decline (Richeldi et al., 2011; Richeldi et al., 2014a; Richeldi et al., 2016a).

Hepatotoxicity in individuals treated with pirfenidone is infrequent and typically manifests as a mild increase in aminotransferases. Elevated liver enzymes during pirfenidone therapy are not uncommon. Analysis of safety across four clinical studies revealed that elevations in alanine aminotransferase (ALT) or aspartate aminotransferase (AST) levels (>3× the upper limit of normal) occurred in 2.7% of patients who received pirfenidone (Valeyre et al., 2014), but there was a case report of drug-induced liver injury (DILI) and acute liver injury in patients who received pirfenidone. (Verma et al., 2017). According to global pharmacovigilance data, drug-induced hepatotoxicity has been associated with nintedanib at a rate of 31.5 events/1,000 person-years (Lasky et al., 2020a). Moreover, a previous study (Valeyre et al., 2014) employed the pharmacovigilance database and data from spontaneously self-reported adverse events to assess the safety profile of nintedanib in patients diagnosed with IPF. Their results did not encompass comprehensive information regarding nintedanib dose modifications or discontinuations and lacked patient comorbidities, patient characteristics and concomitant medication use. Therefore, further studies are necessary to address the safety of pirfenidone and nintedanib in clinical practice. The aim of our study was to evaluate the risk of hepatotoxicity in patients with IPF who received pirfenidone and nintedanib in Taiwan.

## Materials and methods

### Study design and data source

This was a retrospective cohort study, and the data were extracted from the National Health Insurance Research Database (NHIRD). The Taiwan NIH (National Health Insurance) programme, which covers 99.6% of 23 million Taiwanese people and 93% of hospitals, clinics and pharmacies, represents one of the largest and most comprehensive medical population databases in the world. The study encompassed the time frame from 1 January 2016, to 31 December 2020, and received approval from the Institutional Review Board (IRB) of Kaohsiung Medical University Hospital. (KMUHIRB-E(I)-20230175).

#### Study population

**FIGURE 1:**
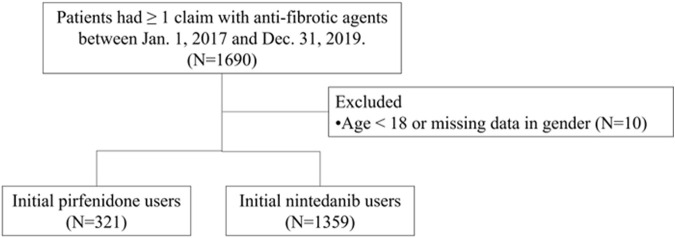
Patient selection flow chart.

Patients with idiopathic pulmonary fibrosis (IPF) were identified using (1) the International Classification of Diseases, 10th Revision, Clinical Modification (ICD-10-CM) diagnosis code J84.1 and (2) at least one medical claim code for antifibrotic agents between 1 March 2017, and 31 December 2019. The date of initial prescription of any antifibrotic medication was designated the index date. The antifibrotic agents used included nintedanib and pirfenidone. Due to the NHI insurance criteria, in which nintedanib or pirfenidone were only selected for use, there were no combination antifibrotic agents in the study population. The exclusion criteria were patients who had used antifibrotic agents prior to the index date, individuals younger than 18 at the index date, and those with incomplete data regarding gender or age. The follow-up period extended until censoring, death, or up to 1 year following the index date, see Figure 1.

#### Baseline characteristics

All sociodemographic information, including age and age group (18-49, 50-74, ≥75 years old) and sex (male or female), was collected. The baseline comorbidities in the IPF cohorts were characterized by the presence of two or more outpatient claims or one or more inpatient claims with ICD-10-CM diagnosis codes for these comorbidities within 1 year before and up to the index date (Supplementary Appendix S1). In this study, the comorbidities included rheumatoid arthritis, systemic lupus erythematosus, dermatomyositis, polymyositis, sicca syndrome, sarcoidosis, pulmonary hypertension, emphysema, chronic obstructive pulmonary disease, pulmonary embolism, lung cancer, hypertension, type 2 diabetes mellitus, sleep apnea, gastroesophageal reflux disease, anxiety, depression, hepatitis B, hepatitis C, nonalcoholic fatty liver disease, nonalcoholic steatohepatitis, alcoholic liver disease, biliary diseases, liver cancer, and liver cirrhosis/fibrosis. The comedications were defined as having ≥28 days within a year before and including the index date; these included antituberculosis agents, antibacterial agents, antifungal agents, nonsteroidal anti-inflammatory drugs, anticonvulsants, statins, antigout agents, antiarrhythmia agents, and proton pump inhibitors (Supplementary Appendix S2).

## Study outcomes

The main outcome was the incidence of drug-induced liver injury (DILI), which was defined as a composite of an inpatient diagnosis of acute liver failure, toxic liver disease with cholestasis, necrosis of the liver, acute hepatitis, hepatomegaly, jaundice, nonspecific elevation of transaminase and lactic acid dehydrogenase levels, liver transplant status, and liver cancer (Supplementary Appendix S3). The secondary outcome included one outpatient or inpatient diagnosis of DILI. The study outcomes were identified during the follow-up time within 30 days, 180 days, and 365 days after the index date.

### Statistical analysis

For the descriptive statistics, the categorical variables are presented as numbers and percentages. The continuous variables are presented as the mean (standard deviation, SD).

Univariate and multivariate Poisson regression analyses with robust error variance were conducted to compute unadjusted and adjusted incidence rate ratios (IRRs) along with 95% confidence intervals (CIs) for each specific outcome. The adjusted variables included sex, age group, comorbidity (hepatitis B virus infection, hepatitis C virus infection, nonalcoholic fatty liver disease, nonalcoholic steatohepatitis, alcoholic liver disease, biliary disease, liver cancer, liver cirrhosis), and comedication (antibiotics, proton pump inhibitors, nonsteroidal anti-inflammatory drugs, HMG-CoA reductase inhibitors). The incidence rate ratios (IRRs) for all study outcomes, along with additional sensitivity analyses, were assessed using the Poisson regression model with robust error variance, distinguishing between nintedanib and pirfenidone. Various sensitivity analyses were performed. First, intention-to-treat and per-protocol analyses were used to ensure that the results were consistent with different medical exposures. The first prescription for nintedanib or pirfenidone on the index date was defined as the medical group. Per-Protocol was defined as the medical group from the first prescription of nintedanib or pirfenidone at the index date to the drop out/stop index drugs, censoring, outcome occurrence, or last date of the follow-up period in the analysis of only those patients who strictly adhered to the protocol. Second, we divided the outcomes into specific DILI codes, nonspecific DILI codes, and liver cancer cases in the nintedanib and pirfenidone groups. Third, we used falls as an outcome to test for negative results, which were not connected with an increased risk with nintedanib or pirfenidone exposure. All the statistical analyses were conducted using SAS 9.4 (SAS Institute, Inc., Cary, North Carolina, United States). These analyses were two-tailed, and a significance level of *p* ≤ 0.05 was regarded as statistically significant.

## Results

All patients who had ≥1 claim with pirfenidone or nintedanib between Jan. 1, 2017, and Dec. 31, 2019, were included in the analysis. We excluded those patients younger than 18 years or those with missing data (N = 10). Finally, 321 patients received pirfenidone, and 1,359 patients received nintedanib. Among the patients who received pirfenidone, the mean age ±standard deviation (SD) was 75.2 ± 10.8 years, 73.8% were male, and 55.1% were aged ≥75 years (Table 1). Among the patients who received nintedanib, the mean age ±SD was 73.8 ± 9.7 years, 76.6% were male, and 49.2% were aged ≥75 years. Patients who received pirfenidone had a greater incidence of comorbidities than patients who received nintedanib for rheumatoid arthritis, systemic lupus erythematosus, dermatomyositis, polymyositis, sicca syndrome, sarcoidosis, pulmonary hypertension, emphysema, chronic obstructive pulmonary disease, hypertension, type 2 diabetes mellitus, nonalcoholic fatty liver disease, hepatitis C virus infection, liver cirrhosis/fibrosis and liver cancer Figure 1.

**TABLE 1:** Patient baseline characteristics.

	Total (N = 1,680)	Initial treatment group	
		Pirfenidone (N = 321)	Nintedanib (N = 1,359)
Male, n (%)	1,278 (76.1)	237 (73.8)	1,041 (76.6)
Age, mean ± SD	74.1 ± 9.9	75.2 ± 10.8	73.8 ± 9.7
Age group, n (%)			
18-49	14 (0.8)	4 (1.2)	10 (0.7)
50-74	821 (48.9)	140 (43.6)	681 (50.1)
≥75	845 (50.3)	177 (55.1)	668 (49.2)
Comorbidity, n (%)			
Rheumatoid arthritis	55 (3.3)	15 (4.7)	40 (2.9)
Systemic lupus erythematosus	19 (1.1)	4 (1.2)	15 (1.1)
Dermatomyositis	7 (0.4)	3 (0.9)	4 (0.3)
Polymyositis	10 (0.6)	3 (0.9)	7 (0.5)
Sicca syndrome	71 (4.2)	17 (5.3)	54 (4.0)
Sarcoidosis	6 (0.4)	2 (0.6)	4 (0.3)
Emphysema	186 (11.1)	37 (11.5)	149 (11.0)
Chronic obstructive pulmonary disease	897 (53.4)	186 (57.9)	711 (52.3)
Pulmonary hypertension	39 (2.3)	8 (2.5)	31 (2.3)
Pulmonary embolism	11 (0.7)	1 (0.3)	10 (0.7)
Lung cancer	107 (6.4)	16 (5.0)	91 (6.7)
Hypertension	915 (54.5)	189 (58.9)	726 (53.4)
Type 2 diabetes mellitus	579 (34.5)	119 (37.1)	460 (33.8)
Sleep apnea	37 (2.2)	5 (1.6)	32 (2.4)
Gastroesophageal reflux disease	418 (24.9)	77 (24.0)	341 (25.1)
Anxiety	171 (10.2)	32 (10.0)	139 (10.2)
Depression	51 (3.0)	7 (2.2)	44 (3.2)
Hepatitis B	51 (3.0)	9 (2.8)	42 (3.1)
Hepatitis C	46 (2.7)	10 (3.1)	36 (2.6)
Nonalcoholic fatty liver disease	21 (1.3)	5 (1.6)	16 (1.2)
Nonalcoholic steatohepatitis	7 (0.4)	1 (0.3)	6 (0.4)
Alcoholic liver disease	14 (0.8)	2 (0.6)	12 (0.9)
Biliary diseases	11 (0.7)	1 (0.3)	10 (0.7)
Liver cancer	26 (1.5)	5 (1.6)	21 (1.5)
Liver cirrhosis/fibrosis	38 (2.3)	10 (3.1)	28 (2.1)
Comedication, n (%)			
Antituberculosis agents	6 (0.4)	1 (0.3)	5 (0.4)
Antibacterial agents	1,213 (72.2)	244 (76.0)	969 (71.3)
Antifungal agents	43 (2.6)	9 (2.8)	34 (2.5)
Nonsteroidal anti-inflammatory drug	1,099 (65.4)	213 (66.4)	886 (65.2)
Anticonvulsants	18 (1.1)	4 (1.2)	14 (1.0)
Statins	624 (37.1)	137 (42.7)	487 (35.8)
Antigout agents	122 (7.3)	27 (8.4)	95 (7.0)
Anti-arrhythmia agents	83 (4.9)	22 (6.9)	61 (4.5)
Proton pump inhibitors	337 (20.1)	67 (20.9)	270 (19.9)

We used intention-to-treat Poisson regression analysis for outcomes identified from inpatient diagnosis, as shown in Table 2. Patients who received nintedanib had a higher risk of DILI from inpatient diagnosis using specific codes after adjusting for variables including sex, age group, comorbidity (nonalcoholic fatty liver disease, hepatitis B virus infection, hepatitis C virus infection, nonalcoholic steatohepatitis, alcoholic liver disease, biliary disease, liver cancer, liver cirrhosis, Epstein–Barr virus disease, and autoimmune hepatitis), and comedication (antibiotics, nonsteroidal anti-inflammatory drugs, HMG-CoA reductase inhibitor, proton pump inhibitors), with an aIRR of 3.62 (95% CI 1.11–11.78). Similarly, the risk of DILI was greater in patients who received nintedanib if we used per-protocol Poisson regression analysis (Table 3) for outcomes identified from inpatient diagnosis using specific codes after adjustment for gender, age group, comorbidity (hepatitis B virus infection, hepatitis C virus infection, nonalcoholic fatty liver disease, nonalcoholic steatohepatitis, alcoholic liver disease, biliary disease, liver cancer, liver cirrhosis, Epstein–Barr virus disease, and autoimmune hepatitis), and comedication (antibiotics, nonsteroidal anti-inflammatory drugs, HMG-CoA reductase inhibitor, proton pump inhibitors) with aIRR 3.60 (95% CI 1.11–11.72).

**TABLE 2:** Intention-to-treat Poisson regression analysis for outcomes identified from inpatient diagnosis.

Intention-to-treat (ITT)	Within 30 days after index date				Within 180 days after index date				Within 365 days after index date			
	Event (N)	Follow-up (Day)	cIRR (95% CI)	aIRR (95% CI)§	Event (N)	Follow-up (Day)	cIRR (95% CI)	aIRR (95% CI)§	Event (N)	Follow-up (Day)	cIRR (95% CI)	aIRR (95% CI)§
Inpatient DILI (specific codes)												
Nintedanib	7	40259	NA	NA	27	222827	2.19 (0.66-7.22)	2.68 (0.80-8.94)	39	419882	3.21 (0.99-10.38)	3.62 (1.11-11.78)*
Pirfenidone	<3	9570	Ref.	Ref.	3	54242	Ref.	Ref.	3	103584	Ref.	Ref.
Inpatient DILI (nonspecific codes)												
Nintedanib	9	40243	NA	NA	36	222165	2.93 (0.90-9.51)	3.33 (1.02-10.87)*	52	418032	3.22 (1.16-8.89)*	3.50 (1.26-9.72)*
Pirfenidone	<3	9570	Ref.	Ref.	3	54242	Ref.	Ref.	4	103401	Ref.	Ref.
Inpatient liver cancer												
Nintedanib	5	40295	1.19 (0.14-10.14)	0.74 (0.06-8.90)	16	223688	0.96 (0.32-2.88)	1.25 (0.35-4.41)	19	422938	0.92 (0.34-2.47)	1.40 (0.43-4.51)
Pirfenidone	<3	9551	Ref.	Ref.	4	53822	Ref.	Ref.	5	102499	Ref.	Ref.
Inpatient fall (negative outcome)												
Nintedanib	<3	40346	NA	NA	8	224324	0.96 (0.20-4.54)	0.93 (0.19-4.46)	17	423392	0.82 (0.30-2.23)	0.83 (0.30-2.27)
Pirfenidone	<3	9570	Ref.	Ref.	<3	54010	Ref.	Ref.	5	102538	Ref.	Ref.

* < 0.05.

§ Adjusted variables: gender, age group, comorbidity (hepatitis B, hepatitis C, nonalcoholic fatty liver disease, nonalcoholic steatohepatitis, alcoholic liver disease, biliary disease, liver cancer, liver cirrhosis, Epstein–Barr virus disease, and autoimmune hepatitis), and comedication (antibiotics, nonsteroidal anti-inflammatory drugs, statins, proton pump inhibitors).

**TABLE 3:** Per-protocol Poisson regression analysis for outcomes identified from inpatient diagnosis.

Per-protocol (PP)	Within 30 days after index date				Within 180 days after index date				Within 365 days after index date			
	Event (N)	Follow-up (Day)	cIRR (95% CI)	aIRR (95% CI)§	Event (N)	Follow-up (Day)	cIRR (95% CI)	aIRR (95% CI)§	Event (N)	Follow-up (Day)	cIRR (95% CI)	aIRR (95% CI)§
Inpatient DILI (specific codes)												
Nintedanib	7	40259	NA	NA	27	221045	2.18 (0.66-7.20)	2.69 (0.81-8.98)	39	410448	3.17 (0.98-10.25)	3.60 (1.11-11.72)*
Pirfenidone	<3	9570	Ref.	Ref.	3	53610	Ref.	Ref.	3	100019	Ref.	Ref.
Inpatient DILI (nonspecific codes)												
Nintedanib	9	40243	NA	NA	36	220383	2.92 (0.90-9.48)	3.33 (1.02-10.90)*	52	408598	3.18 (1.15-8.78)*	3.48 (1.25-9.65)*
Pirfenidone	<3	9570	Ref.	Ref.	3	53610	Ref.	Ref.	4	99836	Ref.	Ref.
Inpatient liver cancer												
Nintedanib	5	40295	1.19 (0.14-10.14)	0.74 (0.06-8.90)	16	221917	0.96 (0.32-2.87)	1.24 (0.35-4.40)	19	413246	0.91 (0.34-2.44)	1.39 (0.43-4.49)
Pirfenidone	<3	9551	Ref.	Ref.	4	53190	Ref.	Ref.	5	98934	Ref.	Ref.
Inpatient fall (negative outcome)												
Nintedanib	<3	40346	NA	NA	8	222530	0.96 (0.20-4.52)	0.93 (0.19-4.43)	17	413532	0.81 (0.30-2.21)	0.81 (0.30-2.23)
Pirfenidone	<3	9570	Ref.	Ref.	<3	53378	Ref.	Ref.	5	98973	Ref.	Ref.

* < 0.05.

§ Adjusted variables: gender, age group, comorbidity (hepatitis B, hepatitis C, nonalcoholic fatty liver disease, nonalcoholic steatohepatitis, alcoholic liver disease, biliary disease, liver cancer, liver cirrhosis, Epstein–Barr virus disease, and autoimmune hepatitis), and comedication (antibiotics, nonsteroidal anti-inflammatory drugs, statins, proton pump inhibitors).

Table 4 shows the intention-to-treat Poisson regression analysis for outcomes identified from outpatient and inpatient diagnoses. The risk of DILI diagnosed with specific codes was greater in patients who received nintedanib than in those who received pirfenidone, with an aIRR of 2.54 (95% CI 1.09–5.92). Table 5 shows the per-protocol Poisson regression analysis for outcomes identified from outpatient and inpatient diagnoses. The risk of DILI diagnosed with specific codes was greater in patients who received nintedanib than in those who received pirfenidone, with an aIRR of 2.54 (95% CI 1.09–5.91).

**TABLE 4:** Intention-to-treat Poisson regression analysis for outcomes identified from outpatient and inpatient diagnosis.

Intention-to-treat (ITT)	Within 30 days after index date				Within 180 days after index date				Within 365 days after index date			
	Event (N)	Follow-up (Day)	cIRR (95% CI)	aIRR (95% CI)§	Event (N)	Follow-up (Day)	cIRR (95% CI)	aIRR (95% CI)§	Event (N)	Follow-up (Day)	cIRR (95% CI)	aIRR (95% CI)§
Outpatient/inpatient DILI (specific codes)												
Nintedanib	17	40184	4.04 (0.54-30.35)	4.01 (0.53-30.37)	45	220554	1.83 (0.78-4.29)	1.99 (0.84-4.70)	59	414596	2.44 (1.05-5.65)*	2.54 (1.09-5.92)*
Pirfenidone	<3	9547	Ref.	Ref.	6	53881	Ref.	Ref.	6	102853	Ref.	Ref.
Outpatient/inpatient DILI (nonspecific codes)												
Nintedanib	23	40092	1.09 (0.41-2.86)	1.02 (0.38-2.73)	65	218469	1.44 (0.76-2.72)	1.47 (0.77-2.81)	85	409186	1.61 (0.90-2.89)	1.62 (0.90-2.92)
Pirfenidone	5	9472	Ref.	Ref.	11	53100	Ref.	Ref.	13	100876	Ref.	Ref.
Outpatient/inpatient liver cancer												
Nintedanib	18	40104	0.85 (0.32-2.29)	1.14 (0.29-4.51)	25	222153	1.00 (0.41-2.44)	1.19 (0.41-3.49)	26	420028	0.90 (0.39-2.07)	1.01 (0.38-2.72)
Pirfenidone	5	9483	Ref.	Ref.	6	53350	Ref.	Ref.	7	101657	Ref.	Ref.
Outpatient/inpatient fall (negative outcome)												
Nintedanib	<3	40346	NA	NA	10	224150	0.80 (0.22-2.91)	0.86 (0.23-3.15)	21	422731	0.73 (0.31-1.71)	0.74 (0.31-1.75)
Pirfenidone	<3	9570	Ref.	Ref.	3	53925	Ref.	Ref.	7	102259	Ref.	Ref.

* < 0.05.

§ Adjusted variables: gender, age group, comorbidity (hepatitis B, hepatitis C, nonalcoholic fatty liver disease, nonalcoholic steatohepatitis, alcoholic liver disease, biliary disease, liver cancer, liver cirrhosis, Epstein–Barr virus disease, and autoimmune hepatitis), and comedication (antibiotics, nonsteroidal anti-inflammatory drugs, statins, proton pump inhibitors).

**TABLE 5:** Per-protocol Poisson regression analysis for outcomes identified from outpatient and inpatient diagnosis.

Per-protocol (PP)	Within 30 days after index date				Within 180 days after index date				Within 365 days after index date			
	Event (N)	Follow-up (Day)	cIRR (95% CI)	aIRR (95% CI)§	Event (N)	Follow-up (Day)	cIRR (95% CI)	aIRR (95% CI)§	Event (N)	Follow-up (Day)	cIRR (95% CI)	aIRR (95% CI)§
**Outpatient/inpatient DILI (specific codes)**												
Nintedanib	17	40184	4.04 (0.54-30.35)	4.01 (0.53-30.37)	45	218772	1.83 (0.78-4.28)	1.99 (0.84-4.72)	59	405162	2.41 (1.04–5.58)*	2.54 (1.09–5.91)*
Pirfenidone	<3	9547	Ref.	Ref.	6	53249	Ref.	Ref.	6	99288	Ref.	Ref.
**Outpatient/inpatient DILI (nonspecific codes)**												
Nintedanib	23	40092	1.09 (0.41-2.86)	1.02 (0.38-2.73)	65	216687	1.43 (0.76-2.71)	1.48 (0.77-2.83)	85	399752	1.60 (0.89-2.86)	1.63 (0.90-2.93)
Pirfenidone	5	9472	Ref.	Ref.	11	52521	Ref.	Ref.	13	97549	Ref.	Ref.
**Outpatient/inpatient liver cancer**												
Nintedanib	18	40104	0.85 (0.32-2.29)	1.14 (0.29-4.51)	25	220382	1.00 (0.41-2.43)	1.19 (0.41-3.48)	26	410336	0.89 (0.39-2.05)	1.01 (0.37-2.71)
Pirfenidone	5	9483	Ref.	Ref.	6	52718	Ref.	Ref.	7	98092	Ref.	Ref.
**Outpatient/inpatient fall (negative outcome)**												
Nintedanib	<3	40346	NA	NA	10	222448	0.72 (0.19-2.65)	0.69 (0.19-2.60)	20	413148	0.68 (0.29-1.61)	0.67 (0.28-1.59)
Pirfenidone	<3	9570	Ref.	Ref.	3	53293	Ref.	Ref.	7	98694	Ref.	Ref.

* < 0.05.

§ Adjusted variables: gender, age group, comorbidity (hepatitis B, hepatitis C, nonalcoholic fatty liver disease, nonalcoholic steatohepatitis, alcoholic liver disease, biliary disease, liver cancer, liver cirrhosis, Epstein–Barr virus disease, and autoimmune hepatitis), and comedication (antibiotics, nonsteroidal anti-inflammatory drugs, statins, proton pump inhibitors).

## Discussion

To our knowledge, this is the first cohort study utilizing a nationwide health insurance database to explore the risk of DILI in patients with IPF who were administered nintedanib and pirfenidone. The results showed that nintedanib was associated with a greater risk of DILI than pirfenidone among patients with IPF after adjusting for comorbidities and medication. The outcomes were consistent across several subgroup analyses between inpatient, outpatient, intent-to-treat Poisson regression, per-protocol Poisson regression, specific codes, or nonspecific codes.

The incidence, prevalence, and mortality rate of IPF have consistently remained high in recent years (Joung et al., 2023). Over time, in treated IPF patients in participating European centers, various therapeutic regimens (acetylcysteine, azathioprine, prednisolone, and mycophenolic acid) have been used in combination with two antifungal drugs (Guenther et al., 2018). An increasing number of studies have started to accumulate on postmarketing surveillance of rare and common adverse outcomes associated with these two antifibrotics drugs. Postmarketing surveillance has been carried out to assess the safety and tolerability of nintedanib in real-world studies. The most frequent adverse event reported in patients who received nintedanib was diarrhea; additional adverse events of special interest included cardiovascular adverse events, abnormal hepatic function and bleeding (Cottin, 2017).

A large-scale postmarketing surveillance study to survey the safety of nintedanib in Japanese patients with pulmonary fibrosis in real-world clinical practice was performed, involving the analysis of 5,578 patients. Overall, 2,795 individuals (50.1%) ceased nintedanib treatment within the first 12 months after initiation, and 67.5% of patients experienced adverse drug reactions (Ogura et al., 2023). The most common adverse drug reactions requiring discontinuation of the drug within 3 and 12 months were elevated liver function (18.8%) and diarrhea (13.2%), respectively (Ogura et al., 2023). These adverse drug reactions were more common in postmarketing surveillance studies than in prospective studies. Integrated analysis of two prospective studies, TOMORROW and INPULSIS, revealed that more than 52 weeks of nintedanib treatment is tolerable and safe in patients with pulmonary fibrosis (Richeldi et al., 2016b). In these two prospective studies, the discontinuation rate for patients receiving nintedanib was 20.6%, whereas it was 15.0% for those treated with a placebo. The most common reason for discontinuation was diarrhea (5.3%), which was lower than what was observed in the postmarketing surveillance study. Importantly, the nintedanib studies did not include pulmonary fibrosis patients with moderate (Child‒Pugh B) or severe (Child‒Pugh C) hepatic dysfunction, and the use of nintedanib is not recommended for these patients. In both of the INPULSIS trials, 4.9% of participants in INPULSIS-1 and 5.2% of participants in INPULSIS-2 exhibited elevated aspartate aminotransferase/alanine aminotransferase levels, or both, exceeding three times the upper limit of normal (Richeldi et al., 2014a). Abnormal liver enzyme levels were not correlated with clinical symptoms or signs of liver injury and could be reversed through dose reduction or discontinuation of nintedanib. An additional study, which utilized the global pharmacovigilance database to evaluate the safety and tolerability of nintedanib in patients with pulmonary fibrosis, revealed abnormal liver enzyme events at a rate of 31.5 events per 1,000 patient-years. Notably, this rate is lower than that of the INPULSIS study (162 events per 1,000 patient-years) (Lasky et al., 2020b). Our data also showed similar results. In the intention-to-treat group, inpatient DILI with a specific code followed up for 365 days showed a rate of 39 events in 498882 patient-days. In the intention-to-treat group, inpatient/outpatient DILI with a specific code followed up for 365 days showed a rate of 59 events in 498882 patient-days. Elevated liver enzymes can occur during the 1-year follow-up in patients who receive nintedanib. The adverse event of DILI reported in the global pharmacovigilance database is in line with our observations from the NHIRD database. DILI can potentially manifest in patients with IPF who are administered nintedanib. It is advisable that liver function tests be conducted prior to the initiation of treatment, followed by regular monitoring of liver enzymes during the first 3 months of treatment, and should continue to be conducted at regular intervals thereafter. (Lasky et al., 2020b). Because DILI can occur in the first year, we recommend periodically checking liver enzymes in the first year or as clinically indicated to minimize the risk of permanent liver injury.

Compared with nintedanib, pirfenidone had a lower risk of liver injury. A prospective nationwide postmarketing study from 10 Korean institutions showed that poor appetite (32.4%) and photosensitivity reactions (13.7%) were the most common adverse events. Of the 258 enrolled patients, 8 (3.7%) had abnormal liver function tests (Chung et al., 2020). Another postmarketing survey of all patients with pulmonary fibrosis in Japan who were administered pirfenidone also showed that common adverse drug reactions were decreased appetite and photosensitivity (Ogura et al., 2015). Post-marketing surveillance data used to survey potential hepatotoxicity from the drug database are limited due to the absence of actual liver enzyme data. Prospective clinical trials of pirfenidone and nintedanib demonstrated lower rates of hepatotoxicity, likely because the information obtained from clinical trial patients may follow a specific DILI diagnosis protocol.

## Limitations

Our study has several limitations. First, the number of patients in the pirfenidone group was lower than that in the nintedanib group. Second, the absence of liver enzyme data limits the interpretability of the results. To address this limitation, we conducted an analysis from various perspectives, including inpatient and outpatient data, specific and nonspecific diagnostic codes, intent-to-treat, and per-protocol approaches. Our findings consistently demonstrated similar results across different analytical methods between the two groups, aligning closely with previous observations. Third, variations in geography and ethnicity may contribute to differences in the incidence of DILI. Further studies are necessary to corroborate these findings and refine the estimates.

## Conclusion

Based on our real-world observations, the risk of DILI associated with nintedanib aligns with what was noted in previous clinical trials and the global pharmacovigilance database. Therefore, routine monitoring of liver enzymes and patient education are advised when initiating nintedanib to mitigate the risk of sustained liver damage. Additionally, further research into the safety and tolerability profile of patients with pulmonary fibrosis receiving antifibrotic treatment is warranted. Post-marketing surveillance data used to survey potential hepatotoxicity from the drug database are limited because the database lacks actual liver enzyme data and because no formal causality assessment was performed. Consequently, so-called real-world usage might overestimate the risk of hepatotoxicity. The diagnosis of DILI requires more information than what an insurance database contains.

## Data Availability

Data are available from the National Health Insurance Research Database (NHIRD) published by Taiwan National Health Insurance (NHI) Bureau. Due to legal restrictions imposed by the government of Taiwan in relation to the “Personal Information Protection Act”, data cannot be made publicly available. Requests for data can be sent as a formal proposal to the NHIRD (http://nhird.nhri.org.tw).
