# Supplementary material for: Risk of potential hepatotoxicity from pirfenidone or nintedanib in patients with idiopathic pulmonary fibrosis: results of a retrospective analysis of a large insurance database in Taiwan
Source: Front Pharmacol. 2024 Feb 7;15:1309712. doi: 10.3389/fphar.2024.1309712 (PMC10879927; doi:10.3389/fphar.2024.1309712)
Supplement: Supplementary file 1 [file DataSheet1.docx]

Appendix

Appendix 1. ICD-10-CM codes for baseline comorbidities

| Comorbidity | ICD-10-CM codes | Comorbidity | ICD-10-CM codes |
| --- | --- | --- | --- |
| Rheumatoid arthritis | M05.7-M05.9, M06.0, M06.2, M06.3, M06.8, M06.9, M08.0-M08.9 | Sleep apnea | G47.3 |
| Systemic lupus erythematosus | M32.x | Gastroesophageal reflux disease | K21 |
| Dermatomyositis | M33.0, M33.1, M33.9, M36.0 | Anxiety | F41 |
| Polymyositis | M33.2 | Depression | F32, F33 |
| Sicca syndrome | M35.0 | Hepatitis B | B16, B17.0, B18.0, B18.1, B19.1 |
| Sarcoidosis | D86.0, D86.2 | Hepatitis C | B17.1, B18.2, B19.2 |
| Emphysema | J43, J98 | Nonalcoholic fatty liver disease | K76.0 |
| Chronic obstructive pulmonary disease | J44 | Nonalcoholic steatohepatitis | K75.81 |
| Pulmonary hypertension | I27.0, I27.2 | Alcoholic liver disease | K70 |
| Pulmonary embolism | I26, I27.82 | Biliary diseases | K83 |
| Lung cancer | C34, D02.2, D14.3, D38.1 | Liver cancer | C22 |
| Hypertension | I10-I13, I15 | Liver cirrhosis / fibrosis | K74 |
| Type 2 diabetes mellitus | E10-E14 |  |  |

Appendix 2. ATC codes for baseline comedications

| Comedication | ATC codes |
| --- | --- |
| Antituberculosis agents | J04AM05 |
| Antibacterial agents | J04AM08, J01EC01, J01EE01, J01CA04, J01CR02, A02BD06, A02BD07, A02BD10, A02BD03, A02BD05, A02BD01, A02BD16, A02BD04, A02BD11, A02BD12, A02BD13, A02BD14, A02BD15, A01AB23, D10AF07, J01AA08, J01DB01, J01DB04, J01FA10, S01AA26, J01RA07, J01RA16, J01MA02, S01AE03, S02AA15, S03AA07, J01RA10, J01RA11, J01RA12, A02BD10, J01MA12, S01AE05, J01RA05, J01DC09, J01DD12, J01DD62, J01CA04, J01CR02, A02BD06, A02BD07, A02BD10, A02BD03, A02BD05, A02BD01, A02BD16, A02BD04, A02BD11, A02BD12, A02BD13, A02BD14, A02BD15, J01DD04, J01DD54, J01DD63, D10AF02, J01FA01, S01AA17, D10AF52, J01CF01, J01CA12, J01CR05, J01DH02, J01DH52, A07AA09, J01XA01, S01AA28, D10AF01, G01AA10, J01FF01, D10AF51, J01DH03 |
| Antifungal agents | D01AC08, G01AF11, H02CA03, J02AB02, D01AE15, D01BA02, J02AC02 |
| Nonsteroidal anti-inflammatory drug | D11AX18, M01AB05, M02AA15, S01BC03, S01CC01, M01AB55, M01AG01, N02AJ08, C01EB16, G02CC01, M01AE01, M02AA13, R02AX02, M01AE51, N02AJ19, C01EB03, M01AB01, M02AA23, S01BC01, S01CC02, M01AB51, M01AH05, C08CA51, L01XX33, M01AH01, N02AJ16, M01AX17, M02AA26 |
| Anticonvulsants | N03AB02, N03AB52, N03AF01, N03AG01, N02BF01, N03AX09 |
| Statins | A10BH52, C10AA07, C10BX05, C10BX09, C10BA06, C10BA09, C10BX16, C10BA07, C10BX17, C10BX10, C10BX07, C10BX14, C10BX13, C10AA04, C10AA05, C10BX08, C10BX03, C10BA05, C10BA08, C10BX15, C10BX12, C10BX06, C10BX19, C10BX11, C10BX18, C10AA01, C10BX01, C10BA02, C10BA04, C10BX04, A10BH51, C10AA03, C10BX02, C10BA11, C10BA03, C10BA12, C10AA02, C10BA01, C10AA08 |
| Antigout agents | M04AA01, M04AA51, M04AA03 |
| Anti-arrhythmia agents | C01BD01, C01BD07 |
| Proton pump inhibitors | A02BC05, A02BD06, M01AE52, A02BC01, A02BD05, A02BD01, A02BD16, A02BC03, A02BD07, A02BD10, A02BD03, A02BD09, A02BC53, A02BD02 |

Appendix 3. ICD-10-CM codes for outcomes

| ICD-10-CM codes | Diagnosis |
| --- | --- |
| Drug-induced liver injury | |
| Specific code |  |
| K71.0 | Toxic liver disease with cholestasis |
| K71.1 | Toxic liver disease with necrosis of the liver |
| K71.2 | Toxic liver disease with acute hepatitis |
| K71.6 | Toxic liver disease with hepatitis, not otherwise classified |
| K71.9 | Toxic liver disease, unspecified |
| K72.0 | Acute and subacute liver failure |
| K72.9 | Liver failure, not specified |
| K75.9 | Inflammatory liver disease, not specified |
| K76.2 | Central hemorrhage necrosis of the liver |
| Nonspecific code |  |
| K76.8 | Other specified diseases of liver |
| K76.9 | Liver disease, unspecified |
| R16.0 | Hepatomegaly, not elsewhere classified |
| R16.2 | Hepatomegaly with splenomegaly, not elsewhere classified |
| R17 | Unspecified jaundice, excludes neonatal |
| R74.0 | Nonspecific elevation of levels of transaminase and lactic acid dehydrogenase |
| Z94.4 | Liver transplant status |
| Other outcomes | |
| C22 | Liver cancer |
| Negative outcome: fall | |
| W00-W19 V00 | Fall |
